# Phylogenetic and Sequence Analyses of the Variable Region in the Glycoprotein Gene of the Respiratory Syncytial Virus Isolated from Iraqi Patients

**DOI:** 10.21315/mjms2024.31.6.11

**Published:** 2024-12-31

**Authors:** Mohammed Hussein Wali, Hassan Mohammad Naif, Nur Arzuar Abdul Rahim, Muhammad Amir Yunus

**Affiliations:** 1 Department of Biomedical Sciences, Advanced Medical and Dental Institute, Universiti Sains Malaysia, Pulau Pinang, Malaysia; 2 Department of Molecular and Medical Biotechnology, College of Biotechnology, Al-Nahrain University, Baghdad, Iraq

**Keywords:** respiratory syncytial virus, attachment glycoprotein, HVR2, PCR, Iraq

## Abstract

**Background:**

Respiratory syncytial virus (RSV) is a common aetiological agent that causes respiratory infections, especially among infants. Identifying circulating RSV genotypes is an essential strategy for understanding the spread of the virus in a certain area. Sequencing the variable regions of the attachment glycoprotein (G) gene of RSV is a quick and direct approach for identifying the genotypes.

**Methods:**

This study was aimed to sequence the G gene region of RSV isolated from patients admitted to hospitals in Baghdad, Iraq, during the autumn of 2022 and winter of 2023. To achieve this goal, 150 patients with lower respiratory symptoms were screened for RSV infections. RSV-positive samples were detected and confirmed using the reverse transcription-quantitative polymerase chain reaction (RT-qPCR) approach, which involved the use of specific TaqMan primer sets targeting RSV subgroups. Then, a G gene region that included hypervariable region 2 (HVR2) was amplified and sequenced using the Sanger sequencing method. Furthermore, molecular and phylogenetic analyses were performed on the G gene region to determine the variability profile of the tested specimens.

**Results:**

There were 41 (26.6%) RSV-positive cases. Of these, the RSV-B subgroup was the most prevalent (82.90%), while the RSV-A subgroup incidence rate was 17.07%. The phylogenetic analysis showed that the RSV-B isolates were related to the BA genotype and shared nucleotide sequence similarities with isolates from India, Australia and the UK. The RSV-A isolates belonged to the ON genotype and had some degree of similarities with isolates from Italy, Tunisia, and France.

**Conclusion:**

Seasonal tracking of the RSV isolates would facilitate a better understanding of virus evolution, viral pathogenesis, and genetic diversity.

## Introduction

Respiratory syncytial virus (RSV) is one of the common respiratory viruses that causes moderate to severe lower pulmonary infections, including pneumonia and bronchitis. This virus has a highly virulent profile in infected individuals, especially children under one year old. It is estimated that 25% of respiratory infections in children are caused by RSV, which belongs to the *Orthopneumoviruses* genus of the *Pneumoviridae* family (1). This family also includes metapneumovirus, which causes severe respiratory infections. The viruses in this family are aetiological agents for several diseases, and many of these illnesses have high morbidity and mortality rates. The transmission routes and pathogenicity vary depending on the virus type (1–3).

RSV’s nonsegmented, single-stranded RNA material consists of 10 genes responsible for encoding 11 proteins involved in the virus replication process (4). Based on the molecular variations in some of its genes, especially in the G gene region, RSV can be classified into two subgroups, i.e., RSV-A and RSV-B (5). The RSV genome is surrounded by a filamentous envelope with attachment glycoproteins (G), fusion (F) proteins, and small hydrophobic (SH) proteins located inside the envelope layer (4, 6). The main role of the G and F proteins is to facilitate viral entry into respiratory epithelial cells during the infection process. The F gene is considered a conserved gene and has more stability than the G gene (7–10). Meanwhile, the variability of certain regions of the G gene plays a crucial role in differentiating between RSV strains (11).

The G protein has around 300 amino acids and three main domains—cytoplasmic, transmembrane, and extracellular—as well as a structure containing two sets of heavily glycosylated mucin-like domains, which play a major role in binding to the host cell (5, 12, 13). Through the O-linked and N-linked sugars, the G protein is glycosylated, which can assist in easily evading immune system defences and any pre-existence immune cells against RSV (14–16). Hypervariable region 1 (HVR1) and hypervariable region 2 (HVR2) in the superficial ectodomain of the G gene are commonly used to differentiate between RSV sub-genotypes. Furthermore, HVR2’s high variability profile can be used to analyse novel RSV strains (14, 17–20).

Molecular genotyping has been successfully used in monitoring and controlling the global spread of RSV. In one study, RSV sequencing was used to identify a new RSV-B genotype by analysing the nucleotide sequence of the G gene region through direct comparison with the global National Center for Biotechnology Information (NCBI) Virus database (21). This finding highlights the importance of viral genome sequencing in identifying RSV genotypes to provide reliable surveillance data on newly emerging RSV cases. In another study, the complete RSV-A genome was sequenced to study the genetic diversity and geographical distribution of RSV subgroups in Saudi Arabia (22). The study findings demonstrated the effectiveness of the sequencing approach in the molecular evaluation of RSV subgroups.

The present study was aimed to determine the RSV subgroups and genotypes circulating in Iraqi children. Samples from 150 patients with lower respiratory symptoms were screened for RSV infection using reverse transcription-quantitative polymerase chain reaction (RT-qPCR) targeting RSV-A and RSV-B. The samples underwent PCR amplification of the G gene region, which included HVR2, and Sanger sequencing analysis. Based on the sequencing results, phylogenetic analysis was conducted to compare the evolution of the local RSV isolates with the global RSV strains retrieved from the NCBI Virus database.

### Repositories

Partial G gene sequences are available at the NCBI under accession numbers OR593487–OR593499.

## Materials and Methods

### Clinical Samples

Nasopharyngeal swab samples were obtained from 150 patients with acute respiratory tract infections (ARTIs) from three hospitals in Iraq during the autumn of 2022 and winter of 2023. The patients suspected of having RSV infection were selected based on their ARTI clinical symptoms and other findings, such as chest X-ray results (if available). All specimens were taken from the patients within 24 hours of admission and up to seven days from the onset of symptoms. Paediatric teaching hospitals, Al- Kadhimiya Educational Hospital and Al-Elwya Hospital in Baghdad were chosen to collect RSV samples from children with ARTIs.

During the sampling process, general data on the patients, including gender, age, period of infection and clinical symptoms, were gathered. The samples were transported to the Molecular Virology Laboratory, College of Biotechnology, Al-Nahrain University, kept in viral transport media (VTM) stored at 4°C for no more than 72 hours, and subjected to RNA extraction.

### Primer Design

#### RSV Detection Using qPCR Targeting the L Gene

The main challenge in RSV detection lies in the variability of the viral gnome. To achieve precise viral detection, a conserved genomic region should be used. The polymerase (L) gene, which encodes for the polymerase enzyme, is typically used as a target sequence for designing a PCR primer set for the polymerase (L) gene. PCR primer sets targeting the L gene was modified by Todd and his colleagues (23) to match all possible RSV genotypes. These primer sets have two probes specifically designed for the RSV-A and RSV-B genotypes that run in a single reaction, enabling the detection of both RSV subgroups.

In the present study, this primer set was examined using the RSV nucleotide database on the NCBI Virus website. Multiple consensus, which were retrieved from a number of RSV sequences in the NCBI Virus database, were generated to match all RSV genotypes. Based on the final sequence alignment, the primer set used by Todd et al. (23) was chosen with some modifications to the forward primer (Table 1).

#### RSV Genotyping Through Conventional PCR Targeting the G Gene

To improve the analysis of the genetic profile and the genotyping of the RSV-positive samples, the primers for the G gene were designed based on the HVR2 region, where the most variability occurs. To enable the sequencing of all G gene areas, especially the gene terminal where HVR2 is located, the reverse primers for both subgroups were in the F gene area, which is next to the G gene. To help produce a phylogenetic profile that could lead to a better understanding of the RSV distribution in Iraq, the G gene sequencing results were compared with global databases found in the NCBI Virus database. Additional molecular analysis was conducted to search for regions in the G gene where there was differentiation between the RSV subgroups. The primer annealing sites were manually selected in conserved area in the G gene for the forward strand and in the F gene for the reverse strand. These primer sets were also used in the sequencing reactions of the PCR amplicons. Details on each primer location, length, and nucleotide sequence are provided in Table 1.

### Viral RNA Isolation and Detection of RSV-positive Samples

RSV’s genomic RNA was automatically isolated using the Maxwell Viral Nucleic Acid Extraction Kit (Promega, WI, US) in accordance with the manufacturer’s instructions. The yield and purity of the isolated RNA were validated using a NanoDrop instrument (Thermo Fisher Scientific, MA, US). All RNA samples were stored at −70°C until additional analysis was conducted.

To identify the RSV-positive samples, the oasigTM lyophilised OneStep RT-qPCR Kit (PrimerDesign, England, UK) was used in accordance with the manufacturer’s instructions and with the primers and probes listed in Table 1. RT-qPCR was performed using the QuantStudio 5 Real-Time PCR instrument (Applied Biosystems, Thermo Fisher Scientific, MA, US). The RSV-positive samples, which had a cycle threshold (Ct) value below 40, were stored at −70°C for further use in complementary DNA (cDNA) synthesis.

### cDNA Synthesis

To generate the G gene-specific cDNA template from the RSV-positive RNA samples, the reverse transcription (RT) assay was performed using the GoScript^TM^ Reverse Transcription Kit (Promega, WI, US) and a SimpliAmp Thermal Cycler (Applied Biosystems, Thermo Fisher Scientific, MA, US) in accordance with the manufacturer’s instructions. Upon completion of the reaction, the purity and concentrations of the cDNA were measured using a NanoDrop device (Thermo Fisher Scientific, MA, US). Samples with high purity and concentrations of cDNA were stored at −70°C for further analysis.

### PCR Amplification of the Targeted G Gene Region of RSV

A region of the G gene of RSV, which had an approximate size of 900 base pair (bp) and included HVR2, was amplified using conventional PCR and a designated set of primer pairs (Table 1). The PCR reaction was performed using the SimpliAmp Thermal Cycler and a cDNA template generated from the RSV-positive samples with a Ct value below 40. The total volume of the reaction was 50 μL, which included 15 μL of master mix, 1.5 μL of forward and reverse primers, 5 μL of the cDNA template (with cDNA concentration of 100–150 ng/μL), and nuclease-free water. The cycling condition involved an initial denaturation at 94°C for 5 min followed by 40 cycles of denaturation at 94°C for 1 min, annealing at 56°C for 30 sec, and extension at 72°C for 30 sec. There was a final extension cycle at 72°C for 5 min, and the resulting PCR product was stored at 4°C for further analysis.

PCR amplicon analysis was conducted using 2% agarose gel electrophoresis. The targeted PCR amplicon band was detected and compared with molecular ladder (DM05-01, Bioland Scientific LLC, CA, US) using the Bio-Rad XR+ Gel Documentation System (Bio-Rad, Watford, UK). Clear and positive PCR amplicon bands that matched the approximate targeted size (around 900 bp) were selected for Sanger nucleotide sequencing.

### Sanger Nucleotide Sequencing of the PCR Amplicons

The Sanger sequencing reaction was performed using the same primer sets as those used to amplify the G gene region (Table 1). Before performing the sequencing reaction, all selected PCR amplicons were subjected to a purification process using an ExoSAP-IT Kit (Applied Biosystems, Thermo Fisher Scientific, MA, US) in accordance with the manufacturer’s instructions. After that, the purified PCR amplicons were sequenced by Macrogen Inc. (Seoul, South Korea) using ABI 3730XL (Applied Biosystems, Thermo Fisher Scientific, MA, US), and the sequencing data were analysed using bioinformatics tools. All nucleotide sequences obtained from the sequencing reaction were individually analysed and cleaned using CLC Workbench (Qiagen, Hilden, Germany) and MEGA 11 software (https://www.megasoftware.net/). Unclear sequence reads at the terminals were trimmed and aligned. In addition, gaps and variable nucleotide regions were examined and saved in the FASTA file format. A selection of nucleotide sequences from some samples, which have some differences in the G gene region, was submitted to the NCBI database.

### Phylogenetic Analysis

The G gene nucleotide sequence data were analysed using CLC Workbench and BioEdit version 7.0.5.3 software (https://bioedit.software.informer.com/7.2/). G gene reference sequences from different geographical regions were retrieved from the NCBI database using the NCBI Virus website. The sequencing data from the present study and the NCBI dataset were compared using the multiple sequence alignment (MSA) tool in the CLC Workbench software. This analysis focused on the G gene region that included HVR2 and detected variations in the nucleotide sequences. The maximum likelihood method was used to construct a phylogenetic tree in the CLC Workbench software based on the MSA data. This tree revealed the similarities and differences between the samples in the present study and the NCBI dataset. Bootstrapping with 100 replicates was performed to evaluate the reliability of the tree’s topology.

### Amino Acid Sequence Analysis

All aligned nucleotide sequences of the targeted RSV G gene location were subjected to codon translation analysis to generate the corresponding amino acid sequences using the standard tool in the CLC Workbench software. Variations in the amino acids of the G gene in the RSV-positive samples were identified by comparing the obtained sequences with the amino acid sequences in the NCBI datasets. MSA enabled the identification of the similarities and differences between the G gene amino acids of the local isolates and similar sequences in the NCBI datasets.

### Statistical Analysis

Statistical analysis was performed using the Statistical Package for the Social Sciences (SPSS) version 20 (IBM Corp., Armonk, NY, US). Patient data (gender, age, time of infection, and clinical symptoms) were statistically compared using the Chi-square test, which analyses the relationship between categorical variables. A *p*-value below 0.05 was considered statistically significant.

## Results

All samples analysed in this study were obtained from patients who were hospitalised for acute lower respiratory tract infections and predominantly exhibited severe influenz-alike symptoms, such as cough, wheezing, fever (above 38°C), chest pain, breathing difficulties, pneumonia, and occasionally, bronchiolitis. Certain patients, notably infants, displayed indications of severe respiratory infections, especially reduced oxygen levels and overall bodily weakness. Furthermore, the majority of the participants had complete blood count results that revealed elevated lymphocyte levels, which are indicative of viral-induced infections.

### Study Cohort Profile and RSV Incidence Rate

**Figure 1:**
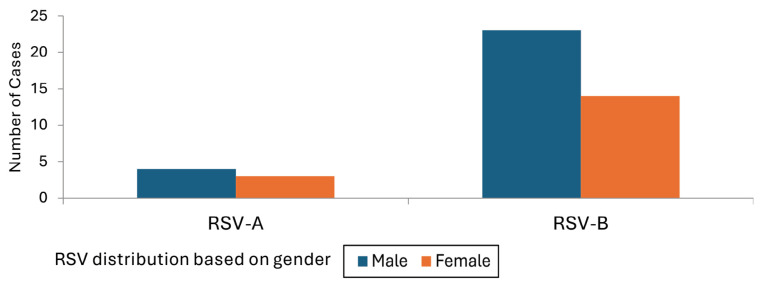
Gender distribution of RSV-positive subtypes A and B among local patients Note: In both RSV subtypes, the male gender is more susceptible to RSV infections across all age groups.

The study population consisted of 105 children under 5 years old (70%) and 45 adults (30%). Regarding gender, 88 (59%) of the participants were male and 62 (41%) were female. The RT-qPCR results confirmed RSV positivity in 41 participants, with 34 cases attributed to RSV-B and 7 to RSV-A. Across all age groups, there was a higher RSV incidence rate among the male participants (24 cases, 58.54%) than the female participants (17 cases, 41.46%), with a *p*-value of 0.033 (Figure 1, Table 2). Regarding age, 32 of the participants with RSV were under 5 years old and 9 were over 25 years old.

### PCR Amplification Results

**Figure 2:**
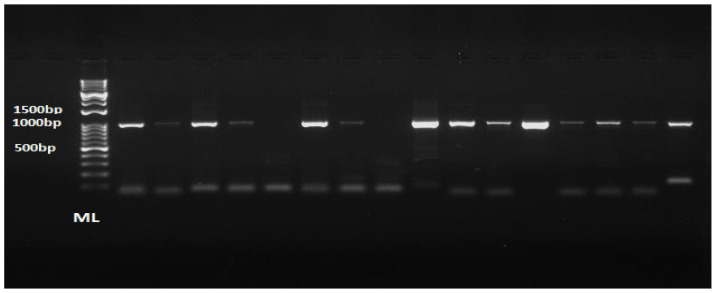
A representative PCR amplification product of the targeted RSV G gene region analysed by gel electrophoresis Notes: Amplicons with the expected size bands (approximately 1000 bp, lanes 1–16) were observed. The size of the G gene bands was measured based on the molecular ladder DM05-01. Similar bands were observed on the rest of the tested samples.

The G gene sequence was successfully amplified from the 41 RSV-positive samples. As shown in Figure 2, the PCR amplicons for the samples produced clear, specific DNA bands visualised on the agarose gel. These PCR amplicons were then sequenced using the Sanger sequencing method.

### Synonymous and Nonsynonymous Nucleotide Changes and Amino Acid Sequence Analysis of the Targeted RSV G Gene Region

The sequencing results were analysed to determine the chromatogram peaks for each sample. The terminals for the sequences with noisy signals were trimmed from both ends to obtain reliable and clear sequences. These sequences were aligned with some RSV G gene sequences from the NCBI Virus database. The alignments presented on the BLAST website (https://blast.ncbi.nlm.nih.gov/Blast.cgi) were used to evaluate the sequencing data for each sample. The nucleotide changes detected in a few locations in the G gene region could be used to identify new variations and the genotypes of the local isolates (Tables 3 and 4).

The BLAST alignments revealed reliable matches with several RSV samples from other countries, which were further examined using phylogenetic analysis. The alignments were created for the RSV-B subgroup based on the MZ515863 reference isolate of the BA genotype and for the RSV-A subgroup based on the OR143167 reference isolate. The JN257693 reference isolate (the ON1 genotype) was also included in the alignments to indicate molecular changes at the genotype level.

Several single nucleotide changes were detected in the RSV-B samples (Table 4). At position 31, cytosine was replaced by thymine, leading to an amino acid change (histidine/tyrosine), which was detected in all of the RSV-B samples and the AY333364 isolate from Spain. At position 46, cytosine was replaced by thymine, which was observed in most of the samples and the MZ515871 isolate from the UK. Other nucleotide changes at positions 48, 49, 50, 56, 133, 136, 140, 148, 171, 175, 187, 194, and 204 were detected in several of the samples and some isolates from Europe and Asia. Nucleotide substitutions at positions 45, 74, 92, 111, 120, 141, 168, 179, 188, 200, and 225 were only seen in the samples, which could indicate that there were new amino acid changes in the G protein.

Table 4 shows the amino acid substitutions found in each position. This table focuses on nucleotide changes that caused nonsynonymous mutations in which amino acids were substituted. The amino acid sequence analysis revealed unique single nucleotide variations in the G gene of the local RSV isolates (Table 3). Some of these variations were observed across isolates from multiple countries, suggesting potential consistency of the detected variations between the samples and selected global isolates.

### Phylogenetic Tree of the Tested RSV Isolates Based on Partial G Gene Nucleotide Sequences

**Figure 3:**
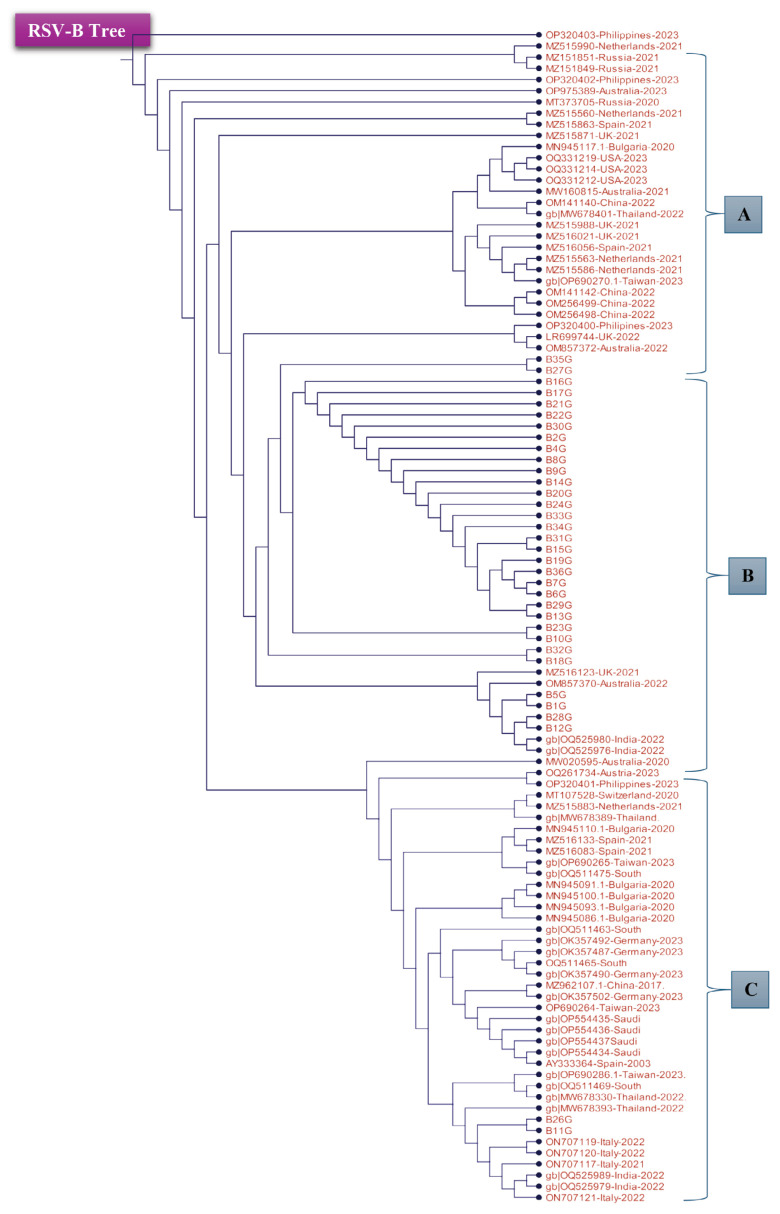
Phylogenetic tree representing the relationship of the RSV-B samples with a selection of NCBI RSV-B isolates based on the sequences of the G gene Notes: The tree was divided into three groups based on the internal branches and nodes. Group A represents the ancestry branch where the other groups (B and C) are generated. The tested RSV-B samples of this study are located within group B.

**Figure 4:**
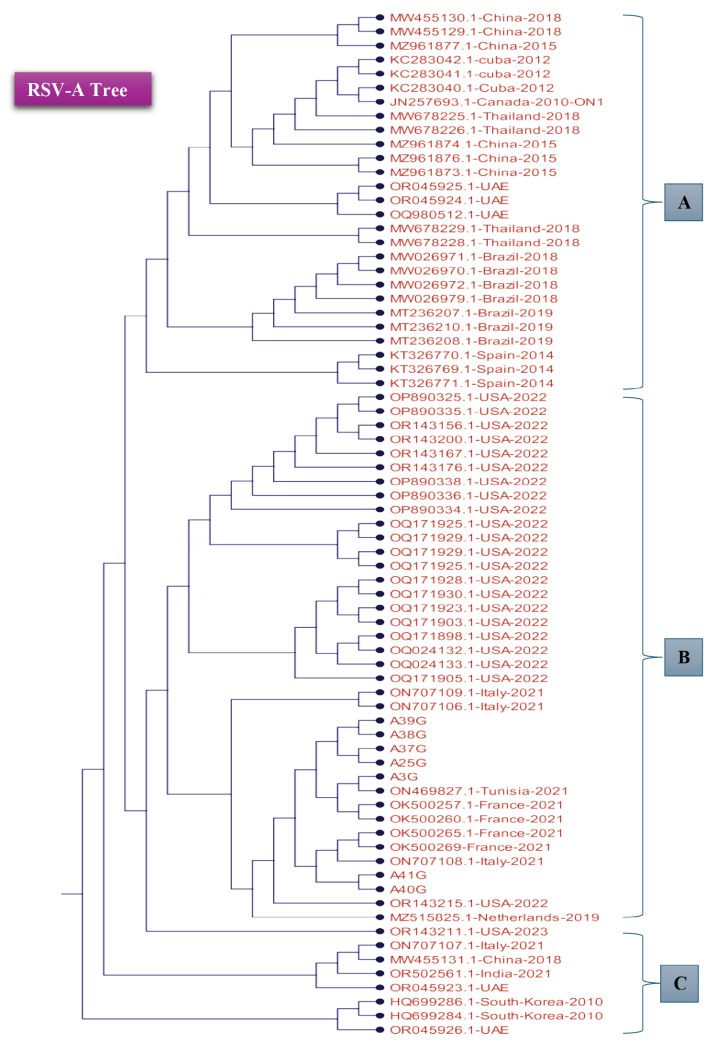
Demonstration of phylogenetic trees constructed between the RSV-A samples and a set of published RSV isolates within the G gene Notes: The tree was divided into three groups based on the ancestry branches where the main node represents group C. The A and B groups were clustered away from the C group. The tested RSV-B samples were located within the A and B groups.

To compare the local and global RSV isolate distributions, 71 established RSV-B isolates of the G gene region were compared with the samples in the present study. A phylogenetic tree was constructed using the neighbour joining algorithm, and the branch distance was measured using the Jukes-Cantor model (Figures 3 and 4). The branch length was used to show the nucleotide change rate between the local and global isolates.

### G Gene Partial Sequence Submission to the NCBI Database

The G gene partial sequences submitted to the NCBI database were examined by the NCBI submission team. Upon sequence verification by the NCBI submission team, the samples were assigned accession numbers between OR593487 and OR593499.

## Discussion

The present study aimed to determine the distribution of RSV cases in several hospitals in Iraq during the autumn of 2022 and winter of 2023 among ARTI hospitalised patients. Molecular and phylogenetic analyses revealed that most of the RSV cases were from the RSV-B subgroup (82.9%); only 7 isolates (17.1%) belonged to the RSV-A subgroup. Similarly, 69% of the samples in the study by Abduljabbar et al. (24) were RSV-B. The prevalence of RSV-B cases in Iraq has been reported in several studies, indicating that this subgroup is frequently circulating in the Middle East region (24–26). However, other studies have identified more cases of RSV-A than RSV-B (27, 28).

The domination of one RSV subgroup over the other might be due to the host immune response, which could make an individual more susceptible to one RSV subgroup. Other factors are the geographical distribution of RSV cases, incident rates according to the season and precautionary measures implemented by the populations (children and elderly patients) during flu seasons and especially during the COVID-19 pandemic. Treggiari et al. (29) found that, after a decrease in the mitigation strategies enforced during the COVID-19 pandemic, the cases of RSV subgroup incidents have been reducing since the end of 2021.

In the present study, several changes were observed in the same locations in HVR2 in the G gene, indicating that similar RSV genotypes are circulating in Iraq. However, there was genetic variability among the studied samples, indicating that distinct RSV genotypes may be circulating among local patients. Thus, genotype (and sub-genotype) identification based on the G gene region was shown to be achievable.

As shown in Figures 3 and 4, the phylogenetic tree was divided into three groups (A, B, and C) based on internal nodes, which had similar sequences emerge from them. The appearance of several nodes from each ancestor node indicated that the coverage area of the targeted nucleotide sequence had some variable nucleotide sequences. This variation was in the tested part of the G gene region, including HVR2. In contrast, in the RSV-B phylogenetic tree (Figure 3), most of the samples emerged from the same node, as predicted.

Meanwhile, some samples showed high degrees of relatedness with isolates from other countries. For instance, samples 1, 5, 12, and 28 had some similarities with OQ525980 and OQ525976, which were isolated in India during the winter of 2022. These samples also shared a similar branch with isolates from the UK (MZ516123, the BA genotype) and Australia (OM857370), which had some nucleotide differences between them and were collected in the autumn and winter of 2021 and 2022, respectively. These findings indicate that there were slight nucleotide changes in the viral genome between autumn and winter seasons. Genetic diversity in the G gene region has also been highlighted in several other studies. For instance, Etemadi et al. (30) revealed the emergence of a new genotype for each RSV subgroup (RSV-A and RSV-B) in Malaysia while focusing on the G gene as the main region for identifying and distinguishing new genotypes in the RSV subgroups.

The phylogenetic analysis showed that the isolates in samples 11 and 26 were branched from a node shared with several reference isolates, including ON707119 from Italy, MW678393 from Thailand, and OQ525989 from India. As in the previous group, nucleotide changes occurred between these samples and the global isolates, as each isolate was collected during different seasons and years and from different countries. The rest of the samples were clustered from the same node, which emerged from similar ancestors of other nodes, including OM857372 from Australia, LR699744 (the BA9 genotype) from the UK, and OP320400 from the Philippines.

The seven samples in the RSV-A phylogenetic tree (Figure 4) were aligned with the NCBI dataset. There was a high degree of similarity in the samples’ nucleotide sequences, as they originated from two nodes where the samples existed. Furthermore, the samples had high rates of matches, which was based on the alignment score, with several global isolates, including ON707106 from Italy, ON469827 from Tunisia, and OK500257, OK500260, and OK500265 from France. They also had similar ancestors with two isolates, i.e., OR143215 from the US and MZ515825 from the Netherlands. This tree was also divided into three regions (A, B, and C) based on the branches from the main node. Regarding the isolates’ distribution in each group, nucleotide changes were observed in the G gene regions of several NCBI isolates from different geographical locations in different seasons. The observed nucleotide changes in this study could also exist in isolates from other countries, as the virus is spreading throughout the world.

There were also novel nucleotide changes in the G gene region in the samples, and 11 of the nucleotide substitutions were nonsynonymous. As shown in Table 4, the amino acid changes in the RSV-B samples occurred at different locations in the G gene and could suggest structural changes in the G protein. These changes could be established as unique markers for the generation of new genotypes, or they could be related to emerging subgenotypes. The existence of such genetic variability in the G protein could help the virus escape the host immune system and/or improve its attachment to host epithelial cells. In addition, the G protein is typically heavily glycosylated, which prevents its identification by host antibodies (15, 31).

The study findings also revealed a higher prevalence of RSV infection in male participants, especially among children less than 5 years of age. This finding has also been reported in previous studies (14, 24, 29, 32, 33). Additionally, the RSV infection is potentially higher in males less than five years of age than in other age groups. This finding is consistent with the observation by Yassine et al. (26) of links between age and gender and the prevalence of the RSV infection. Yassine et al. (26) conducted a systematic review that revealed valuable data on RSV distribution across different seasons and viral genotypes and associations with gender and age factors in Middle Eastern countries, including Iraq. However, there was a rather small amount of information on Iraq due to low number of retrieved data on the RSV cases and genotypes before 2020. Hassan et al. (25) found that RSV cases were potentially high in infants, especially in males, relative to other respiratory viruses. Many studies have highlighted the coinfections of RSV with other viruses and the important role of these coinfections play in causing acute and severe illnesses (33–36).

## Conclusion

Surveillance data acquisition on the RSV cases in each season and year is paramount to understanding the virus’s behaviour and eventually controlling its spread. The study findings showed that RSV-positive cases were still relatively high (27%), while the other cases could have been related to other respiratory viruses or bacteria. The phylogenetic trees developed from the data revealed a high prevalence of the BA genotype (82%) in the RSV-B subgroup and the ON1 genotype (71%) in the RSV-A subgroup. The segregation of the nucleotide sequences in some parts of the G gene region in the global NCBI databases indicates the genetic diversity of the RSV, which helps the virus evade the host immune system and causes pulmonary infections, especially in children. Therefore, genetic variations in the RSV genome, particularly in the G region, should be monitored to help limit the spread of RSV infections and potentially support vaccine development against new RSV genotypes.

## Figures and Tables

**Table 1:** Primer pairs used for RSV detection and genotyping targeting the L and G genes

No.	Primer name	Sequence	Gene name	Location	*Tm	Product length
1	Real-time PCR	**F:** AATACAGMMAARTCYAAYCAACTTTAYA	L	13,850	59.8	94
		**R:** GCCAAGGAAGCATGCARTARA		13,943	57.7	

		**RSV-A-probe:** CYTTARTRCACAATAGCA	L	13,899	#	
		**RSV-B-probe**: GACATCYTTAGTAAGGAAYAGTG		13,905		

2	RSV-A-G	**F:** CATCATATTCATAGCCTCGGCA	G	4,861	58.0	*941–1014
		**R:** GAGCACTAAGATAGCCTTTGCT		5,802	58.0	

3	RSV-B-G	**F:** CATCATCTCTGCCAATCACAAAG	G	4,872	58.0	*922–977
		**R:** ACCTCTGCTAACTGCACTACATG		5,794	60.0	

Notes:

* The product length for some primers is given as range values due to the high variability profile, especially in the G gene area; Tm = melting temperature. The sequence, location, and product length of primers were based on the RSV reference sequences with accession numbers NC_038235.1 and NC_001781.1 for RSV-A and RSV-B, respectively.

**Table 2:** The distribution of study population according to gender, age and symptoms

Variable	No. of patients (total = 150)	Frequency (%)	*p*-value
Male	88	59	0.033
Female	62	41	0.033
Age > 5 years old	105	70	0.012
Age < 5 years old	45	30	0.012
Fever	130	87	0.001
Cough	120	80	0.001
Wheezing	53	35	0.040
Breathing difficulties	41	27	0.020
Pneumonia	23	15	0.029

Note: *p*-value was calculated for each variable based on the total study population using the Chi-square test.

**Table 3:** Details of the single nucleotide changes detected within the G gene of the RSV-B virus

No.	Position on MSA	Variation	Codon changes	Amino acid substitution	Note
1	34	A>G	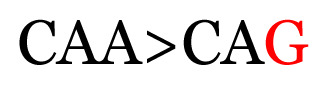	Q>Q	Synonymous mutation
2	53	A>G	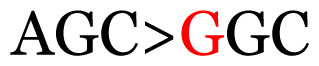	S>G	Nonsynonymous mutation
3	76	C>T	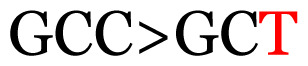	A>A	Synonymous mutation
4	79	A>T	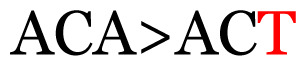	T>T	Synonymous mutation
5	89	C>T	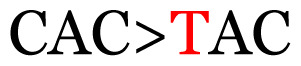	H>Y	Nonsynonymous mutation
6	131	A>G	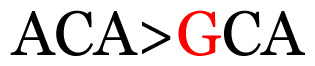	T>A	Nonsynonymous mutation
7	134	C>T	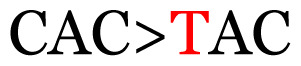	H>Y	Nonsynonymous mutation
8	141	A>C	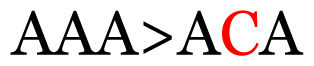	K>T	Nonsynonymous mutation
9	165	T>C	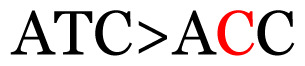	I > T	Nonsynonymous mutation
10	218	C>T	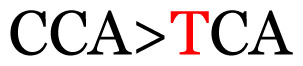	P>S	Nonsynonymous mutation
11	223	A>T	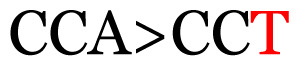	P>P	Synonymous mutation
12	272	A>G	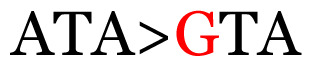	I > V	Nonsynonymous mutation
13	304	T>C	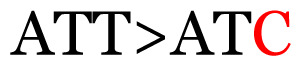	I > I	Synonymous mutation
14	325	T>C	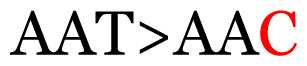	N>N	Synonymous mutation
15	330	C>T	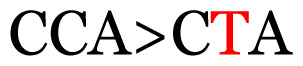	P>L	Nonsynonymous mutation
16	357	C>T	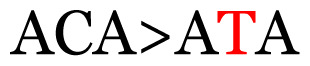	T>I	Nonsynonymous mutation
17	370	T>C	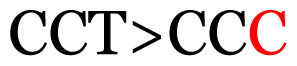	P>P	Synonymous mutation
18	394	C>T	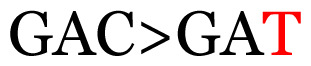	D>D	Synonymous mutation
19	395	C>T	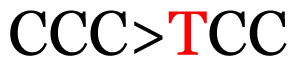	P>S	Nonsynonymous mutation
20	405	T>C	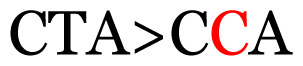	L>P	Nonsynonymous mutation
21	417	C>T	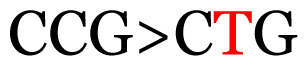	P>L	Nonsynonymous mutation
22	419	A>G	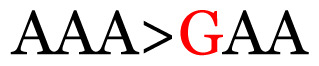	K>E	Nonsynonymous mutation
23	441	C>T	CCA>CTA	P>L	Nonsynonymous mutation
24	460	C>T	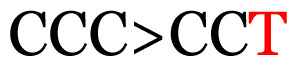	P>P	Nonsynonymous mutation
25	500	G>A	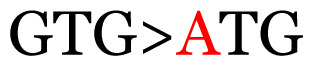	V>M	Nonsynonymous mutation
26	510	T>C	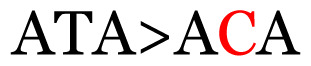	I>T	Nonsynonymous mutation
27	520	A>T	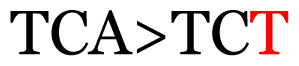	S>S	Synonymous mutation
28	523	A>G	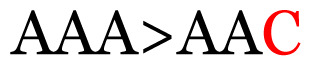	K>N	Nonsynonymous mutation
29	534	G>A	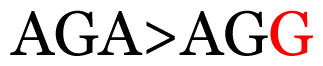	R>K	Nonsynonymous mutation
30	558	T>C	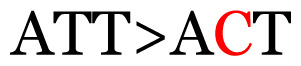	I > T	Nonsynonymous mutation
31	560	G>A	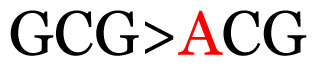	V>T	Nonsynonymous mutation
32	565	T>C	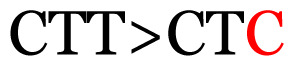	L>L	Synonymous mutation
33	578	T>C	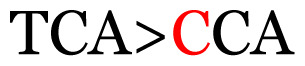	S>P	Nonsynonymous mutation
34	586	C>T	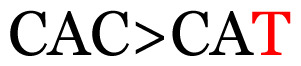	H>H	Synonymous mutation
35	596	C>A	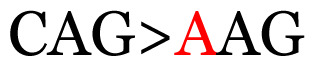	Q>K	Nonsynonymous mutation
36	608	T>C	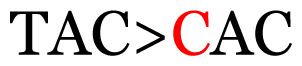	Y>H	Nonsynonymous mutation
37	640	C>T	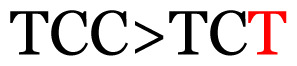	S>S	Synonymous mutation
38	661	C>T	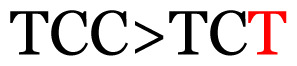	S>S	Synonymous mutation
39	672	C>A	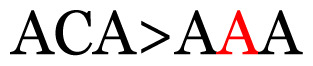	T>K	Nonsynonymous mutation

Notes: The column “Position” refers to the nucleotide location on each variation based on the nucleotide sequences of the tested samples. The nucleotides labelled with red colour represent the synonymous or nonsynonymous changes. The type of each variation in the last column (“Note”) is written based on the detected variations in each codon.

**Table 4:** Amino acid variations and substitutions found in the G gene of the RSV-B isolates from local patients.

No.	Position	Codon in NCBI isolates	Amino acid symbol (name)	Codon in samples	Amino acid symbol (name)
1	19	GGC	G (glycine)	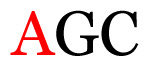	S (serine)
2	31	CAC	H (histidine)	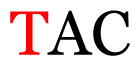	Y (tyrosine)
3	45	ACA	T (threonine)	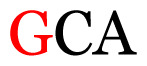	A (alanine)
4	46	CAC	H (histidine)	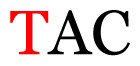	Y (tyrosine)
5	48	ACA	T (threonine)	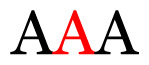	K (lysine)
6	49	ACA	T (threonine)	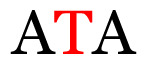	I (isoleucine)
7	50	ACA	T (threonine)	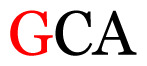	A (alanine)
8	56	ATC	I (isoleucine)	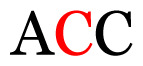	T (threonine)
9	74	CCA	P (proline)	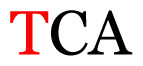	S (serine)
10	92	ATA	I (isoleucine)	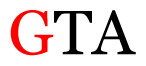	V (valine)
11	111	CCA	P (proline)	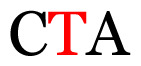	L (leucine)
12	120	ACA	T (threonine)	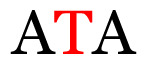	I (isoleucine)
13	133	CCC	P (proline)	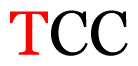	S (serine)
14	136	CTA	L (leucine)	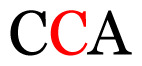	P (proline)
15	140	CCG	P (proline)	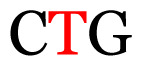	L (leucine)
16	141	AAA	K (lysine)	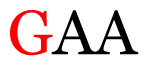	E (glutamic acid)
17	148	CCA	P (proline)	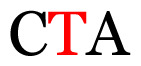	L (leucine)
18	168	GTG	V (valine)	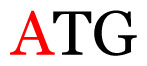	M (methionine)
19	171	ATA	I (isoleucine)	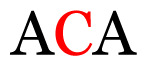	T (threonine)
20	175	AAA	K (lysine)	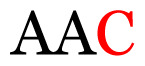	N (asparagine)
21	179	AGG	R (arginine)	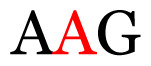	K (lysine)
22	187	ATT	I (isoleucine)	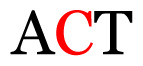	T (threonine)
23	188	GCG	A (alanine)	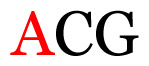	T (threonine)
24	194	TCA	S (serine)	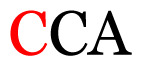	P (proline)
25	200	CAG	Q (glutamine)	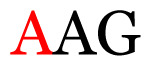	K (lysine)
26	204	TAC	Y (tyrosine)	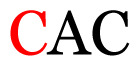	H (histidine)
27	225	ACA	T (threonine)	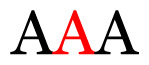	K (lysine)

Note: Nucleotides with red colour refer to the substitutions that occurred in a certain position based on the alignment analysis with a set of NCBI RSV isolates from the amino acid dataset.

## References

[1] Brooks GF, Carroll KC, Butel JS, Morse SA, Mietzner TA (2013). Jawetz, Melnick, & Adelberg’s medical microbiology.

[2] Kutter JS, de Meulder D, Bestebroer TM, van Kampen JJA, Molenkamp R, Fouchier RAM (2021). Small quantities of respiratory syncytial virus RNA only in large droplets around infants hospitalized with acute respiratory infections. Antimicrob Resist Infect Control.

[3] Suryadevara M, Domachowske JB (2021). Epidemiology and seasonality of childhood respiratory syncytial virus infections in the tropics. Viruses.

[4] Pandya MC, Callahan SM, Savchenko KG, Stobart CC (2019). A contemporary view of respiratory syncytial virus (RSV) biology and strain-specific differences. Pathogens.

[5] Bergeron HC, Tripp RA (2021). Immunopathology of RSV: An updated review. Viruses.

[6] Shang Z, Tan S, Ma D (2021). Respiratory syncytial virus: From pathogenesis to potential therapeutic strategies. Int J Biol Sci.

[7] Tabor DE, Fernandes F, Langedijk AC, Wilkins D, Lebbink RJ, Tovchigrechko A (2021). Global molecular epidemiology of respiratory syncytial virus from the 2017–2018 INFORM-RSV study. J Clin Microbiol.

[8] Zhu Q, McLellan JS, Kallewaard NL, Ulbrandt ND, Palaszynski S, Zhang J (2017). A highly potent extended half-life antibody as a potential RSV vaccine surrogate for all infants. Sci Transl Med.

[9] Chen X, Xu B, Guo J, Li C, An S, Zhou Y (2018). Genetic variations in the fusion protein of respiratory syncytial virus isolated from children hospitalized with community-acquired pneumonia in China. Sci Rep.

[10] Saito M, Tsukagoshi H, Sada M, Sunagawa S, Shirai T, Okayama K (2021). Detailed evolutionary analyses of the f gene in the respiratory syncytial virus subgroup A. Viruses.

[11] Tabatabai J, Prifert C, Pfeil J, Grulich-Henn J, Schnitzler P (2014). Novel respiratory syncytial virus (RSV) genotype ON1 predominates in Germany during winter season 2012–13. PLoS ONE.

[12] Hause AM, Henke DM, Avadhanula V, Shaw CA, Tapia LI, Piedra PA (2017). Sequence variability of the respiratory syncytial virus (RSV) fusion gene among contemporary and historical genotypes of RSV/A and RSV/B. PLoS ONE.

[13] Tapia LI, Shaw CA, Aideyan LO, Jewell AM, Dawson BC, Haq TR (2014). Gene sequence variability of the three surface proteins of human respiratory syncytial virus (HRSV) in Texas. PLoS ONE.

[14] Korsun N, Angelova S, Trifonova I, Voleva S, Grigorova I, Tzotcheva I (2021). Predominance of ON1 and BA9 genotypes of respiratory syncytial virus (RSV) in Bulgaria, 2016–2018. J Med Virol.

[15] Efstathiou C, Abidi SH, Harker J, Stevenson NJ (2020). Revisiting respiratory syncytial virus’s interaction with host immunity, towards novel therapeutics. Cell Mol Life Sci.

[16] Ruzin A, Pastula ST, Levin-Sparenberg E, Jiang X, Fryzek J, Tovchigrechko A (2018). Characterization of circulating RSV strains among subjects in the OUTSMART-RSV surveillance program during the 2016–17 winter viral season in the United States. PLoS ONE.

[17] Aamir UB, Salman M, Nisar N, Badar N, Alam MM, Ansari J (2020). Molecular characterization of circulating respiratory syncytial virus genotypes in Pakistani children, 2010–2013. J Infect Public Health.

[18] Almajhdi FN, Farrag MA, Amer HM (2014). Group B strains of human respiratory syncytial virus in Saudi Arabia: Molecular and phylogenetic analysis. Virus Genes.

[19] Ha Do LA, Wilm A, van Doorn HR, Lam HM, Sim S, Sukumaran R (2015). Direct whole-genome deep-sequencing of human respiratory syncytial virus A and B from Vietnamese children identifies distinct patterns of inter- and intra-host evolution. J Gen Virol.

[20] Vandini S, Biagi C, Lanari M (2017). Respiratory syncytial virus: The influence of serotype and genotype variability on clinical course of infection. Int J Mol Sci.

[21] Muñoz-Escalante JC, Comas-García A, Bernal-Silva S, Noyola DE (2021). Respiratory syncytial virus B sequence analysis reveals a novel early genotype. Sci Rep.

[22] Al-Sharif HA, El-Kafrawy SA, Yousef JM, Kumosani TA, Kamal MA, Khathlan NA (2020). Dominance of the on1 genotype of RSV-A and ba9 genotype of RSV-B in respiratory cases from Jeddah, Saudi Arabia. Genes.

[23] Todd AK, Costa AM, Waller G, Daley AJ, Barr IG, Deng YM (2021). Rapid detection of human respiratory syncytial virus A and B by duplex real-time RT-PCR. J Virol Methods.

[24] Abduljabbar HL, Hussein AA, Al-Mayah QS, Aufi IM (2019). Phylogenetic analysis of respiratory syncytial virus isolated from children with respiratory tract infections in Baghdad City, Iraq. J Phys Conf Ser.

[25] Hassan DA, Rachid SK, Ziebuhr J (2018). A single-center study of viral respiratory tract infections in hospitalized children from the Kurdistan region of Iraq. *Glob Pediatr Health*.

[26] Yassine HM, Sohail MU, Younes N, Nasrallah GK (2020). Systematic review of the respiratory syncytial virus (RSV) prevalence, genotype distribution, and seasonality in children from the middle east and North Africa (MENA) region. Microorganisms.

[27] Jumma SS, Jarullah BAH (2022). Molecular study for respiratory syncytial virus in Thi-Qar province, Iraq. Univ Thi-Qar J Sci.

[28] Naji ZM, Abbas MD, Naif HM, Hassani HH, Alhamdani FG (2016). Molecular characterization of fusion and glycoprotein genes of RSV genotype a in infants suffering from influenza like symptoms in Iraq. Int J Sci Technol.

[29] Treggiari D, Piubelli C, Formenti F, Silva R, Perandin F (2022). Resurgence of respiratory virus after relaxation of COVID-19 containment measures: A real-world data study from a regional hospital of Italy. Int J Microbiol.

[30] Etemadi MR, Sekawi Z, Othman N, Lye MS, Moghaddam FY (2013). Circulation of human respiratory syncytial virus strains among hospitalized children with acute lower respiratory infection in Malaysia. Evol Bioinform.

[31] Vigerust DJ, Shepherd VL (2007). Virus glycosylation: Role in virulence and immune interactions. Trends Microbiol.

[32] Al-Romaihi HE, Smatti MK, Al-Khatib HA, Coyle PV, Ganesan N, Nadeem S (2020). Molecular epidemiology of influenza, RSV, and other respiratory infections among children in Qatar: A six years report (2012–2017). Int J Infect Dis.

[33] Na’amnih W, Kassem E, Tannous S, Kagan V, Jbali A, Hanukayev E (2022). Incidence and risk factors of hospitalisations for respiratory syncytial virus among children aged less than 2 years. Epidemiol Infect.

[34] Al-Hussaniy HA, Altalebi RR, Albu-Rghaif AH, Abdul-Amir AGA (2022). The use of PCR for respiratory virus detection on the diagnosis and treatment decision of respiratory tract infections in Iraq. J Pure Appl Microbiol.

[35] Tan J, Wu J, Jiang W, Huang L, Ji W, Yan Y (2021). Etiology, clinical characteristics and coinfection status of bronchiolitis in Suzhou. BMC Infect Dis.

[36] Toh T-H, Hii K-C, Fieldhouse JK, Ting J, Berita A, Nguyen TT (2019). High prevalence of viral infections among hospitalized pneumonia patients in equatorial Sarawak, Malaysia. Open Forum Infect Dis.

